# A comparison between two recommendations to conduct and report systematic reviews on drug’s safety

**DOI:** 10.1186/s13643-019-1167-5

**Published:** 2019-10-16

**Authors:** Ana Penedones, Carlos Alves, Francisco Batel Marques

**Affiliations:** 1 Centre for Health Technology Assessment and Drug Research (CHAD), Association for Innovation and Biomedical Research on Light and Image (AIBILI), Azinhaga Sta. Comba, Celas, 3000-548 Coimbra, Portugal; 2 Laboratory of Social Pharmacy and Public Health, School of Pharmacy, University of Coimbra, Coimbra, Portugal

**Keywords:** Drug-related side effects and adverse reactions, Guideline, Systematic review

## Abstract

**Background:**

Several recommendations are available to conduct and report a systematic review of adverse drug reactions. This study is aimed at identifying and comparing the methodologies of the two most commonly used recommendations to conduct and report systematic reviews on drug’s safety.

**Methods:**

Two systematic reviews were conducted following the recommendations “Cochrane Handbook for Systematic Reviews of Interventions” and “Systematic Reviews’ Centre for Reviews and Dissemination guidance for undertaking reviews in healthcare.” The methods of each recommendation were characterized, and the results and the discussion of each systematic review were also evaluated.

**Results:**

The methodologies of both recommendations are similar. The review question was structured. Both recommendations suggest to include pre- and post-marketing data. The recommended data sources differed and, consequently, the results of the systematic reviews (37 vs. 35 studies). Other aspects of search literature were identical. Different tools are suggested to evaluate the methodological quality of the included studies. For case reports, both recommendations only report some questions that may be helpful to assess risk of bias. The reporting of the results and discussion is also identical for both recommendations.

**Conclusions:**

Few methodological differences were observed between the analyzed recommendations to conduct a systematic review on drug’s safety. Combining their methods into a single and recognized recommendation could be of great value.

## Background

A systematic review can constitute an important tool in pharmacovigilance [1]. A rigorous methodology is used to systematically summarize the available evidence on drug’s safety [2]. Data on the common and expected adverse drug reactions can be obtained from clinical trials [3]. Observational studies, case reports, and spontaneous reports are valuable to detect rare and/or long-term adverse drug reactions [4]. Combining this information using a systematic methodology, which identifies, selects, and critically appraises all available evidence, can expand and strengthen drug’s safety profile [1]. Healthcare professionals, patients, and also regulatory authorities can keep up to date and make informed decisions [5].

To conduct and/or report a systematic review, a defined methodology should be a priori selected. The choice of the recommendation to conduct and/or to report a systematic review is dependent on the review question. For instance, if the aim of a systematic review is to study drug’s efficacy or safety, health economics, and diagnostic test accuracy, among others, the methods will be specific and applied to each area [6–8]. There are different recommendations to conduct and/or report a systematic review [9]. Nevertheless, each recommendation presents different methodology [9]. In a previous study, between 88 and 99% of the recommendations offer guidance on methods (from eligibility criteria to data analysis) [9]. However, some aspects, such as elaboration of an a priori protocol, definition of background, interpretation and discussion of the results, and the need and time for updating the systematic review, presented several discrepancies [9].

Three recommendations are available to conduct and report a systematic review of drug’s safety [6–8]. Each one was developed by an organization created to study and develop guidance on the best synthesis of different types of information [6–8]. The “Cochrane Handbook for Systematic Reviews of Interventions,” developed by the Cochrane Collaboration and last updated on March 2011 [6], and the “Systematic Reviews’ Centre for Reviews and Dissemination guidance for undertaking reviews in healthcare,” developed by the Centre for Reviews and Dissemination (CRD) on 2009 [7], were the recommendations most used to conduct and to report systematic reviews in this field [9]. In 2016, the Preferred Reporting Items of Systematic Reviews and Meta-Analyses (PRISMA) group also developed a guideline to reporting systematic reviews of adverse drug reactions [5].

This study is aimed at identifying and comparing the methodology of the two most commonly used recommendations to conduct and report a systematic review of adverse drug reactions.

## Methods

In a previous work, we had identified three recommendations (“Cochrane Handbook for Systematic Reviews of Interventions,” “Systematic Reviews’ CRD guidance for undertaking reviews in healthcare,” and “Methods Guide for Effectiveness and Comparative Effectiveness Reviews” developed by the Agency for Healthcare Research and Quality (AHRQ)) used to conduct and/or report a systematic review of adverse drug reactions [9]. The recommendations developed by the Cochrane Collaboration [6] and the CRD [7] are the most commonly used and were included to study their methodology. We did not include the recommendation developed by the AHRQ. Although it can be applied to conduct systematic reviews on drug’s safety, this guidance is specific for comparative effectiveness reviews of interventions under the Effective Health Care Program [8].

The two chosen recommendations were named as “A” for “Cochrane Handbook for Systematic Reviews of Interventions” [6] and “B” for “Systematic Reviews’ CRD guidance for undertaking reviews in healthcare” [7]. We conducted two systematic reviews addressing the methodologies defined by the recommendations A and B. Two authors performed both systematic reviews independently, while a third author validated all methodology and results.

At first, a search strategy was defined between consensus among the three authors and replied in each database. The selection of studies and extraction of results were conducted by two authors independently. Disagreements were resolved by discussion and consensus with the third author. The results in each systematic review were analyzed using descriptive analysis and, when it is possible, using meta-analysis. Details of the elaboration of each systematic review are available in the Additional file 1. At the end, the methods of each recommendation were characterized and evaluated. We categorized their methods into “Introduction,” which included “Background,” “Eligibility criteria,” and “Review question”; “Identifying evidence,” including “Type of studies,” “Databases,” “Search strategy,” “Data selection,” “Data extraction,” “Quality assessment,” and “Data synthesis”; and “Reporting,” describing the “Flowchart,” “Characteristics of studies,” “Outcome analysis,” “Quality assessment,” “Discussion,” “Conclusion,” “Funding,” and “Appendix.” Afterwards, a qualitative (exploratory) comparison between the results and impact of these results was performed.

In order to study the influence of different recommendations to conduct a systematic review, the same research question was studied in both systematic reviews. A case study was used to compare both methodologies. We assessed the development of an ophthalmic adverse drug reaction after a suspected medicine exposure. Three regulatory agencies issued a safety alert on the association of non-arteritic anterior ischemic optic neuropathy (NAION) with phosphodiesterase type 5 (PDE_5_) inhibitors [10]. In order to study such association, we defined a research hypothesis, structured according to PICO strategy: to assess the risk of developing NAION in individuals taking PDE5 inhibitors. The results of both systematic reviews can be found in Additional file 1. Herein, the results of the systematic reviews were compiled into a table.

## Results

The methodology suggested by the recommendations A and B to conduct and report a systematic review of adverse drug reactions is summarized in Table 1. We highlighted the sections of the systematic review which differed between both recommendations.

**Table 1:** Summary of methodology used in each systematic review

Step/review	A—Cochrane Collaboration	B—Centre for Reviews and Dissemination
Introduction		
Background	Description of the condition, description of the intervention, how the intervention might work, why it is important to do this research.	Description of intervention, description of the condition, rationale for review.
Eligibility criteria	-Type of participants: patients for whom a PDE_5_ inhibitor is indicated in one of the three approved therapeutic indications. -Type of interventions: PDE_5_ inhibitors (avanafil, lodenafil, mirodenafil, sildenafil, tadalafil, udenafil, and vardenafil) comparing with placebo, active treatment, or no treatment. -Type of outcome measures: development of NAION.	-Population: patients for whom a PDE_5_ inhibitor is indicated in one of the three approved therapeutic indications. -Intervention: PDE_5_ inhibitors (avanafil, lodenafil, mirodenafil, sildenafil, tadalafil, udenafil, and vardenafil). -Comparators: placebo, active treatment, or no treatment. -Outcomes: development of NAION.
Review question	PICO strategy: to assess the risk of NAION associated with PDE5 inhibitors exposure. A systematic review is carried out based on pre- and post-marketing data.	PICO strategy: the objective of this systematic review is to assess the risk of NAION associated with PDE5 inhibitors exposure, based on pre- and post-marketing data.
Identifying evidence		
Type of studies	Randomized controlled trials (RCT), cohort studies, case-control studies, case reports or series of cases, and spontaneous reports.	Randomized controlled trials (RCT), cohort studies, case-control studies, case reports or series of cases, and spontaneous reports.
** Databases**	MEDLINE, EMBASE, Cochrane Controlled Register of Trials (CENTRAL), TRIP*, SCOPUS*, Google Scholar, Web of Science, Open Grey, International Clinical Trials Register Platform, and VigiBase.	MEDLINE, EMBASE, Toxline, Pharmline*, websites of the manufacturers of drugs, and VigiBase.
Search strategy	Search terms comprised the drug name (including the pharmacotherapeutic class, international non-proprietary name (INN), and brand name) and the ophthalmic adverse drug reaction term. A combination of thesaurus terms and free terms was used. No filters were applied to the literature search.	Search terms comprised the drug name (including the pharmacotherapeutic class, international non-proprietary name (INN), and brand name) and the ophthalmic adverse drug reaction term. A combination of thesaurus terms and free terms was used. No filters were applied to the literature search.
Data selection	Two researchers independently screened by hand the titles and abstracts and selected full articles for inclusion.	Two researchers independently screened by hand the titles and abstracts and selected full articles for inclusion.
Data extraction	Data was extracted from each included study by two researchers independently.	Data was extracted from each included study by two researchers independently.
** Quality assessment**	**Included studies were independently assessed for bias according to the methods described in Chapter 13.5 and Chapter 14.6 of the Cochrane Handbook for Systematic Reviews of Interventions.**	**For observational studies, the checklist proposed by Downs and Black was used.** **The case reports were evaluated according to the questions elaborated on the Chapter 4 of the CRD’s guidance for undertaking reviews in health care.**
Data synthesis	Data analysis followed the guidelines set out in Chapter 9 of the Cochrane Handbook for Systematic Reviews of Interventions.	Data from case and spontaneous reports were analyzed using descriptive statistics. A meta-analysis was conducted to analyze data from observational studies.
Reporting		
** Flowchart**	**A predefined flowchart was used.** **37 studies were included in the review, 4 observational studies and 33 case reports (and 608 spontaneous reports).**	**The PRISMA flowchart was used.** **35 studies were included in the review, 4 observational studies and 31 case reports (and 608 spontaneous reports).**
Characteristics of studies	A descriptive table was elaborated. The following information was extracted: reference, country, study design, population (number and demographic data), intervention (and comparator), number of individuals with the ophthalmic adverse drug reaction, risk factor, and medical history.	A descriptive table was elaborated. The following information was extracted: reference, country, study design, population (number and demographic data), intervention (and comparator), number of individuals with the ophthalmic adverse drug reaction, risk factor, and medical history.
Outcome analysis	A meta-analysis was conducted to assess observational studies. A descriptive statistic was used for case reports and spontaneous reports.	A meta-analysis was conducted to assess observational studies. A descriptive statistic was used for case reports and spontaneous reports.
Quality assessment	A table describing the results of risk of bias assessment was developed.	A table describing the results of risk of bias assessment was developed.
Discussion	Summary of main results, overall completeness and applicability of evidence, potential biases in the review process, agreements and disagreements with other studies or reviews.	Principal findings, comparison with other research, strengths and weaknesses of the research.
Conclusion	Implications for practice/research.	Recommendations/implications for practice/further research.
Funding	A financial disclosure was described.	A financial disclosure was described.
Appendix	Search strategy, list of included and excluded studies, Vigibase results, characteristics of studies and quality assessment results.	Search strategy, list of included and excluded studies, Vigibase results, quality assessment results.

^*^Databases not accessible to the authors of the reviews

In general, the methodology of both recommendations is similar. Both systematic reviews provide a detailed rationale for conducting the review, focusing on the description of the intervention and condition, and their possible association. The review question was structured and follows the PICO (Population, Intervention, Comparator, and Outcome) strategy. The two recommendations suggest to search studies including pre- and post-marketing data on drug’s safety. The bibliographic databases and other sources to search evidence suggested in each recommendation differed. Other aspects of search literature, such as the search strategy, data selection, data extraction, and data synthesis, were identical. To evaluate the methodological quality of the included studies, the Cochrane Collaboration developed three scales which evaluate randomized controlled trials, cohort, and case-control studies. The CRD’s guideline (B) recommends some tools to evaluate the several types of studies. For case reports, both guidelines report some questions that may be helpful to evaluate the risk of bias/quality of the reports. The reporting of the results and discussion is also identical for both recommendations.

## Discussion

There are three recommendations to conduct systematic reviews of adverse drug reactions [9]. In order to assess the methodological differences between those recommendations, in this study, two systematic reviews were conducted following the two most commonly used recommendations. Similar approaches in the elaboration of the two systematic reviews of adverse drug reactions were observed.

One of the characteristics that distinguish systematic reviews from narrative reviews is a structured review question [7, 8]. In both systematic reviews, a structured question was presented. It predisposes to a focused hypothesis and can also be useful to define eligibility, search, and inclusion criteria, and presentation of results [11]. Similar to efficacy systematic reviews, systematic reviews focusing on drug’s safety could present a broad or narrow review question [11]. A systematic review studying the association of a class of medicines with a specific adverse drug reaction (for example, the development of retinal detachment after fluoroquinolones exposure [12]) has a narrow focus, while a systematic review studying all adverse drug reactions associated with a class of medicines (for example, the safety profile of ophthalmic anti-angiogenesis inhibitors [13]) has a broad focus [11].

Both recommendations suggested the selection of several types of evidence. These included experimental and observational data. Depending on the review question, each type of sources of information could be the most appropriate. Clinical trials are a robust source of information [3]. Data on common and anticipated adverse drug reactions could be obtained from these studies [3]. Observational studies have some flaws subject of bias in their methodology, such as in the demographic characteristics of the included populations, the follow-up time durations, or the effect size measures used [4]; however, they can provide relevant information on the common, rare, and long-term adverse drug reactions [4]. Examples of observational data include clinical studies in clinical practice, health-administrative database studies, cases and series of case reports, and spontaneous reports [4]. An example of the importance of observational data on drug’s safety assessment is visible in the regulatory decisions. Some safety alerts were issued based only on spontaneous reports [10, 14]. In general, systematic reviews of drug’s efficacy contain data from the highest level of evidence, namely randomized and controlled clinical trials [15]; however, in systematic reviews focusing on drug’s safety, several types of evidence should be included, in order to obtain a more complete and robust drug safety profile. The combination of several types of evidence in safety is already used to make better informed decisions [14]. In 2012, the US Food and Drug Administration issued a safety alert on statins and cognitive side effects. The evidence supporting the regulatory evaluation consisted on randomized controlled trials, observational studies (cohort studies, case-control studies, cross-sectional studies), case reports, and a review of post-marketing spontaneous report database. Thereafter, the adverse drug reaction section of the medicines was updated [14].

A difference which impacts the results of these two systematic reviews was the selection of the bibliographic databases and other sources of information. The authors of the present study only searched in the suggested sources of each recommendation. We also experienced some difficulties in accessing some bibliographic databases. Several studies pointed out the importance of including data from a wide range of bibliographic databases, not only because of their limitations (some databases are more accurate and include newest studies than others), but also because some studies may only be available in one bibliographic database (for instance gray literature is not easily available) [16, 17]. At the present work, we observed some discrepancies between the two systematic reviews in terms of the number of included studies. For instance, two case reports were not included in one of the performed systematic reviews. Nonetheless, we studied the risk of development of a rare adverse drug reaction. Therefore, the volume of available information could be reduced compared with that of a common adverse drug reaction. If the information not found was of higher methodological quality than case reports (such as case-control or cohort studies), the impact of not including this data would be substantial. For instance, in both systematic reviews, the same four observational studies were found. A meta-analysis was conducted based on their results. If a study was missed, the results of this meta-analysis would be different, and consequently, the risk estimate could vary for opposite meanings (risk vs. no risk). Conducting a search using all available sources of information is important, as this will ensure that all relevant data are obtained and evaluated [11]. A systematic review performed by Baudard et al. evaluated the impact of searching on clinical trials registries for additional studies [18]. By analyzing a predefined sample of systematic reviews, this research group found that 52% did not report a search on clinical trials registries [18]. After performing searches on clinical trial registries, Baudard et al. found 122 additional randomized controlled trials [18]. If these studies had been included in meta-analysis, the weight of studies would be changed to almost 60% in some systematic reviews [18]. Other study conducted by Franco et al. found that 73% of reviews have issues in the definition of literature searches [19].

The methodological quality assessment for experimental and observational studies, such as cohort and case-control studies, is possible due to a variety of available tools. However, few or no tools are available to assess other types of observational studies, case reports, health-administrative database studies, case and series of case reports, and spontaneous reports [20]. Since a hierarchy of evidence is not yet defined to assess drug’s safety, several studies questioned the usefulness of these tools once a combination of several types of studies is included in a systematic review and an evaluation of all is not possible [21]. In addition, the majority of the available risk of bias tools are not prepared to assess studies on adverse drug reactions [22]. A systematic review evaluating the quality of reporting in systematic reviews of drug’s safety studies found that a large proportion of the analyzed systematic reviews failed in reporting risk of bias assessment [23]. Nevertheless, some efforts are being made to improve the methodological quality assessment of studies reporting drug’s safety. In addition, a recent study has described a new risk of bias tool to use when conducting systematic reviews of randomized controlled trials, cohort studies, case-control studies, and nested case-control studies describing adverse drug reactions [22].

Some studies assessed the quality of systematic reviews reporting adverse drug reactions. In general, systematic reviews reporting adverse drug reactions failed methodologically [23–25]. Definition of adverse drug reaction, design of literature search, bibliographic database choice, and assessment of methodological quality of the included studies are the main divergent steps [23–25]. In most of the studies, only a small proportion has good reporting [23–25]. In a previous work, we analyzed the methodology used in systematic reviews reporting ophthalmic adverse drug reactions and found the same methodological issues [26]. In 2016, the PRISMA group developed guidance to help reporting systematic reviews of adverse drug reactions, the PRISMA Harms [11]. A study performed by Li et al. evaluated the methods of a sample of systematic reviews, 1 year after the publication of PRISMA Harms [27]. They concluded that a large number of systematic reviews still presented methodological differences [27]. The reinforcement of the use of recommendations to conduct and/or report a systematic review of adverse drug reactions still continues of major importance.

### Limitations of this study

This study has some limitations. Only two recommendations were used to perform a comparison on methods of reviewing drug’s safety data. The two systematic reviews were conducted by the authors of the present study. Only an exploratory analysis was performed to compare both recommendations. A statistical analysis of the methodologies of both recommendations will be necessary to better understand the differences among them. In the two systematic reviews, the authors only searched on the recommended bibliographic databases, despite the recommendations suggested that other databases could be included. Therefore, two case reports were not included in one systematic review. This resulted in an example of the non-inclusion of all available evidence and, consequently, differences in the results of the systematic reviews.

### Further investigation

Further consideration should be taken on the access of the data, including public bibliographic databases and the selection of all available databases, resulting in a more robust and complete data and, therefore, improving the knowledge provided by the systematic review.

In addition, new tools able to evaluate methodological quality of some studies, such as some type of observational studies and case reports, should be elaborated. Moreover, several sources of information should be recorded and more investigation should be performed to clarify the role of the methodological quality assessment in the context of evaluating the evidence of safety.

Finally, the process of conducting and reporting a systematic review of adverse drug reactions, including the design of the review, data search, selection, extraction, and synthesis, should be transparent and independent.

## Conclusions

Few methodological differences were observed among the available recommendations to conduct a systematic review of adverse drug reactions. Combining their methods into a single and recognized recommendation could be of great value. A unique, objective, and easy to apply methodology could improve systematic review’s role in drug safety. Further research should be considered, namely in granting access to the information and in the methodological quality assessment of the included evidence.

## Supplementary information

**Additional file 1.** Results of systematic reviews conducted and reported according to the two most used recommendations on drug’s safety systematic reviews.

## Data Availability

Data sharing is not applicable to this article as no datasets were generated or analyzed during the current study.

## Abbreviations

AHRQ: Agency for Healthcare Research and Quality
CRD: Centre for Reviews and Dissemination
NAION: Non-arteritic ischemic optic neuritis
PDE5: Phosphodiesterase type 5
PICO: Population, Intervention, Comparator, Outcome
PRISMA: Preferred Reporting Items for Systematic Reviews and Meta-Analysis
