# Supplementary material for: A comparison between two recommendations to conduct and report systematic reviews on drug’s safety
Source: Syst Rev. 2019 Oct 16;8:238. doi: 10.1186/s13643-019-1167-5 (PMC6796334; doi:10.1186/s13643-019-1167-5)
Supplement: Supplementary file 1 — Additional file 1. Results of systematic reviews conducted and reported according to the two most used recommendations on drug’s safety systematic reviews. [file 13643_2019_1167_MOESM1_ESM.pdf]

Appendix

Results of systematic reviews conducted and reported according to the two most used recommendations on drug's safety systematic reviews

Step/ Review	A – Cochrane Collaboration	B – Centre for Reviews and Dissemination
Title	Risk of non-arteritic ischemic optic neuropathy with phosphodiesterase type 5 inhibitors: a systematic review and meta-analysis	Risk of non-arteritic ischemic optic neuropathy with phosphodiesterase type 5 inhibitors: a systematic review and meta-analysis
Introduction		
Background	Description of the condition: Ischemic optic neuropathies are the main cause of acute optic nerve injury in Caucasian patients aged 50 years or older.[1-4] Depending on the affected nerve, they can be divided into anterior or posterior ischemic optic neuropathy.[3,4] Ischemic optic neuropathies can also be classified, according to etiology, into arteritic or non-arteritic.[1-4]<br>The pathophysiology of non-arteritic anterior ischemic optic neuropathy (NAION) remains unknown.[1-3] The hypothesis most accepted is that NAION results from small vessel disease, such as an occlusion, of the short posterior ciliary arteries, which supplied the optic nerve head, resulting in hypoperfusion and infarction of the anterior optic nerve.[1-3]<br>Several factors increase the risk of developing NAION.[1-4] Anomalies in optic nerve anatomy, increased age and genetic predisposition, underlying systemic diseases, such as hypertension, episodic hypotension, hypercholesterolemia, diabetes mellitus, prothrombotic states, obstructive sleep apnea, prolonged surgical procedures, cataract surgery, and medication, such as amiodarone, interferon-α, nasal decongestants, several vasopressors or vasoconstricting drugs, and phosphodiesterase type 5 (PDE5) inhibitors.[1-4]<br>The diagnosis of NAION is essentially clinical. NAION is, generally, presented as sudden, painless, and associated with any pattern of visual field loss.[1-4] Patients may present decreased visual acuity, reduced color vision, visual field defect, or flame-shaped haemorrhages.[2] In the fellow eye, small or absent physiological cup may also happen.[1,3]<br>Description of the intervention: The PDE5 inhibitors are a drug class mainly approved for the treatment of erectile dysfunction. Avanafil, lodenafil, mirodenafil, sildenafil, tadalafil, vardenafil and udenafil are examples of	Description of intervention: The phosphodiesterase type 5 (PDE5) inhibitors are a drug class mainly approved for the treatment of erectile dysfunction. Avanafil, lodenafil, mirodenafil, sildenafil, tadalafil, vardenafil and udenafil are examples of selective PDE5 inhibitors. Some of PDE5 inhibitors were also approved for the treatment of signs and symptoms of benign prostatic hyperplasia (tadalafil) and pulmonary arterial hypertension (sildenafil and tadalafil).[1] Sildenafil was the first PDE5 inhibitor introduced in the market, in 1998.[2]<br>The PDE5 enzyme potentiates nitric oxide cascade and concentration of cyclic guanosine monophosphate in the smooth muscle cells, resulting in muscle relaxation, increased blood flow, and prolonged erection[1,3], reverse pulmonary artery remodeling and a reduced pulmonary vascular tone[4,5], and modulate the afferent nerve activity, responsible for the regulation of micturition reflex[6,7].<br>The PDE5 inhibitors are well tolerated and most of their adverse reactions are adjacent to their vascular role.[8] Patients taking nitrate compounds should not use PDE5 inhibitors, since it can result in a sudden hypotension.[8] Headache, flushing, nasal congestion, and dyspepsia are the most common adverse reactions associated with PDE5 inhibitors.[1,3,8] In addition, tadalafil was also related with myalgia and back pain.[8] Patients using PDE5 inhibitors also experienced visual abnormalities, such as changes in color perception, blurred vision and non-arteritic anterior ischemic optic neuropathy (NAION).[1,3]<br>Description of the condition: The development of NAION is, generally, presented as sudden, painless, and associated with any pattern of visual field loss.[9-12] Patients may present decreased visual acuity, reduced color vision,

Step/ Review	A – Cochrane Collaboration	B – Centre for Reviews and Dissemination
	selective PDE5 inhibitors. Some of PDE5 inhibitors were also approved for the treatment of signs and symptoms of benign prostatic hyperplasia (tadalafil) and pulmonary arterial hypertension (sildenafil and tadalafil). [5] Sildenafil was the first PDE5 inhibitor introduced in the market, in 1998.[6]<br>Erectile dysfunction is defined as the inability to achieve or maintain an erection able to satisfactory sexual performance.[7] PDE5 enzyme, found in the smooth muscle of the corpus cavernosum, stimulate hydrolysis of cyclic guanosine monophosphate (cGMP) into GMP, decreasing the concentration of cGMP and nitric oxide (NO) cascade and, consequently, the erection.[5,7] PDE5 inhibitors bind to PDE5 enzymes, avoiding cGMP hydrolysis.[5,7] Therefore, it potentiates NO cascade and concentration of cGMP in the smooth muscle cells in corpus cavernosum, resulting in muscle relaxation, increased blood flow and prolonged erection.[5,7,8]<br>The same mechanism of action is observed for the treatment of pulmonary arterial hypertension and signs and symptoms of benign prostatic hyperplasia.[11-14] PDE5 inhibitors play a role in reverse pulmonary artery remodeling and a reduced pulmonary vascular tone and in the micturition and prostate functioning. PDE5 inhibitors.[11-14]<br>The PDE5 inhibitors are well tolerated and most of their adverse reactions are adjacent to their vascular role.[8] Patients taking nitrate compounds should not use PDE5 inhibitors, since it can result in a sudden hypotension.[8] Headache, flushing, nasal congestion, and dyspepsia are the most common adverse reactions associated with PDE5 inhibitors.[5,7,8] In addition, tadalafil was also related with myalgia and back pain.[5,7] Some serious and rare adverse reactions have been described to PDE5 inhibitors, such as priapism (painful erections), sudden hearing loss and visual abnormalities, such as changes in color perception, blurred vision and NAION.[5,7]<br>How the intervention might work: The association between the use of PDE5 inhibitors and the development of NAION remains unknown.[5,7,10,15] PDE5 inhibitors increase concentration of NO, prolonging vasodilation. This led to a rapid systemic hypotension, one of the risk factors of NAION.[10,15] PDE5 inhibitors may also have a role in the perfusion of optic nerve head, causing a	visual field defect, or flame-shaped hemorrhages.[10] Few patients, almost 10%, reported pain and headache.[9-12] Nevertheless, the pathophysiology of NAION remains unknown.[9-11] The hypothesis most accepted is that NAION results from small vessel disease, such as an occlusion, of the short posterior ciliary arteries, which supplied the optic nerve head, resulting in hypoperfusion and infarction of the anterior optic nerve.[9-11]<br>Several factors increase the risk of developing NAION, such as anomalies in optic nerve anatomy like optic nerve head drusen and small cup-to-disc ratio or absence of the cup; increased age and genetic predisposition; underlying systemic diseases like hypertension, episodic hypotension, hypercholesterolemia, diabetes mellitus, prothrombotic states, obstructive sleep apnea, and blood loss; prolonged surgical procedures; cataract surgery; and medication like amiodarone, interferon-α, nasal decongestants, several vasopressors or vasoconstricting drugs, and PDE5 inhibitors.[9-12]<br>Rationale for review: NAION causes a serious visual disability with sudden vision. PDE5 inhibitors are the first line treatment for erectile dysfunction, which is a common medical condition. Several studies assessed the association between PDE5 inhibitors intake and the development of NAION. A systematic review and meta-analysis can combine all available evidence and provide a more precise result, helpful to healthcare professionals, patients and, also, regulatory authorities.

Step/ Review	A – Cochrane Collaboration	B – Centre for Reviews and Dissemination
	local deregulation.[10,15] PDE6 enzyme is present in ocular blood vessels and have an important function in phototransduction. It is thought that PDE5 inhibitors also act on PDE6, being responsible for changes in color perception.[8,10]<br>Why it is important to do this research: NAION causes a serious visual disability with sudden vision. PDE5 inhibitors are the first line treatment for erectile dysfunction, which is a common medical condition. Several studies assessed the association between PDE5 inhibitors exposure and the development of NAION. A systematic review and meta-analysis can combine all available evidence and provide a more precise result, helpful to healthcare professionals, patients and, also, regulatory authorities.	
Eligibility criteria	-Type of participants: Patients for whom a PDE_5 inhibitor is indicated in one of the three approved therapeutic indications;<br>-Type of interventions: PDE_5 inhibitors (avanafil, lodenafil, mirodenafil, sildenafil, tadalafil, udenafil and vardenafil) comparing with placebo, active treatment or no treatment;<br>-Type of outcome measures: Development of NAION.	-Population: Patients for whom a PDE_5 inhibitor is indicated in one of the three approved therapeutic indications;<br>-Intervention: PDE_5 inhibitors (avanafil, lodenafil, mirodenafil, sildenafil, tadalafil, udenafil and vardenafil);<br>-Comparators: Placebo, active treatment or no treatment;<br>-Outcomes: Development of NAION.
Review question	PICO Strategy: To assess the risk of NAION associated with PDE5 inhibitors exposure. A systematic review is carried out based on pre- and post-marketing data.	PICO Strategy: The objective of this systematic review is to assess the risk of NAION associated with PDE5 inhibitors exposure, based on pre- and post-marketing data.
Identifying evidence		
Type of studies	Randomized controlled trials (RCT), cohort studies, case-control studies, case reports or series of cases and spontaneous reports.	Randomized controlled trials (RCT), cohort studies, case-control studies, case reports or series of cases and spontaneous reports.
Databases	MEDLINE, EMBASE, Cochrane Controlled Register of Trials (CENTRAL), TRIP*, SCOPUS*, Google Scholar, Web of Science, Open Grey, International Clinical Trials Register Platform, and VigiBase.	MEDLINE, EMBASE, Toxline, Pharmline*, websites of the manufacturers of drugs and VigiBase.
Search strategy	Search terms comprised the drug name [including the pharmacotherapeutic class, international non-proprietary name (INN) and brand name] and the ophthalmic adverse drug reaction term. A combination of thesaurus terms and free terms were used. No filters were applied to the literature search. The databases were searched since its inception until November 19, 2018.	Search terms comprised the drug name [including the pharmacotherapeutic class, international non-proprietary name (INN) and brand name] and the ophthalmic adverse drug reaction term. A combination of thesaurus terms and free terms were used. No filters were applied to the literature search. The databases were searched since its inception until November 19, 2018.

Step/ Review	A – Cochrane Collaboration	B – Centre for Reviews and Dissemination
Data selection	Two researchers independently screened by hand the titles and abstracts and selected full articles for inclusion.	Two researchers independently screened by hand the titles and abstracts and selected full articles for inclusion.
Data extraction	Data was extracted from each included study by two researchers independently.	Data was extracted from each included study by two researchers independently.
Quality assessment	Included studies were independently assessed for bias according to the methods described in Chapter 13.5 and Chapter 14.6 of the Cochrane Handbook for Systematic Reviews of Interventions.	For observational studies, the checklist proposed by Downs and Black was used.<br>The case reports were evaluated according to the questions elaborated on the Chapter 4 of the CRD's guidance for undertaking reviews in health care.
Data synthesis	Data analysis followed the guidelines set out in Chapter 9 of the Cochrane Handbook for Systematic Reviews of Interventions.	Data from case and spontaneous reports were analyzed using descriptive statistics. A meta-analysis was conducted to analyze data from observational studies.
Reporting		
Flowchart	A total of 295 potentially relevant records were yielded from literature search (MEDLINE, EMBASE and CENTRAL). Additionally, 462 records were identified through other resources (Google Scholar, Web of Science, Open Grey, International Clinical Trials Register Platform). Two potential articles were identified through reference lists of reviews. Based on above inclusion criteria, 87 records were selected for full-text further inclusion. A final sample of 37 references covering 4 observational studies, 3 series of cases reports and 30 case reports met the inclusion criteria. The selection of references is shown in Figure 1. The references of the included and excluded studies are listed in the Appendix 2. The results of the VigiBase search for NAION events were described below.	A total of 293 potentially relevant publications were yielded from literature search (MEDLINE and EMBASE). Additionally, 61 records were identified through other resources (Toxline). Four potential articles were identified through reference lists of reviews. Based on above inclusion criteria, 77 records were selected for full-text further inclusion. A final sample of 35 references covering 4 observational studies, 3 series of cases reports and 28 case reports met the inclusion criteria. The selection of references is shown in Figure 1. The references of the included and excluded studies are listed in the Appendix 2. The results of the VigiBase search for NAION events reported with PDE5 inhibitors were described below.
Characteristics of studies	Studies: No clinical trials were identified. Four observational studies evaluating the association of PDE5 inhibitors with NAION were identified. Three studies were retrospective. One observational study used the case-control design and two studies were case-crossover. Two studies included patients from United States (USA) in their evaluations.<br>Three series of case reports comprising 22 case reports along with 30 case reports describing the development of NAION when the patient was exposed to a PDE5 inhibitor were identified. Twenty case reports were from USA. A single publication reported 10 case reports from Saudi Arabia.	No RCT were identified. Four observational studies evaluating the association of PDE5 inhibitors with NAION were identified (Table 1). Three studies were retrospective. One observational study used the case-control design and two studies were case-crossover. Two studies included patients from United States (US) in their evaluations. All observational studies evaluated males treated for erectile dysfunction. Their mean age was 64.1 years old. A total of 5,396,708 men were included in the 4 studies. From these, 480,700 were exposed to a PDE5 inhibitor and 4,915,781 men were the comparator. From the total of participants, 114 men were their own control in case-crossover studies. Risk

Step/ Review	A – Cochrane Collaboration	B – Centre for Reviews and Dissemination
	In VigiBase, 689 spontaneous reports of "Eye disorders" were identified (Appendix 3).<br>Participants: All observational studies evaluated males treated for erectile dysfunction. Their mean age was 64.1 years old. A total of 5,396,708 men were included in the 4 studies. 480,700 were exposed to a PDE5 inhibitor and 4,915,781 men were the comparator. From the total of participants, 114 men were their own control in case-crossover studies. Risk factors to develop NAION and medical history were recorded in three studies.<br>A total of 52 patients exposed to a PDE5 inhibitor with NAION were described in the literature. Forty-seven (90%) patients were men. The average age of the patients were 52.9 years old (min= 7 months; max= 76). Twelve (23%) patients had not risk factors to develop NAION. Hypertension (n=16; 31%), diabetes mellitus (n=12; 23%) and dyslipidemia (n=11; 21%) were the most described risk factors.<br>Interventions: All observational studies evaluated the use of PDE5 inhibitors for the treatment of erectile dysfunction. In two studies, the PDE5 inhibitors were specified to vardenafil, tadalafil and sildenafil.<br>Forty (77%) case reports described patients treated for erectile dysfunction, and five (10%) case reports described patients treated for pulmonary arterial hypertension. Sildenafil was the PDE5 inhibitor most reported (n=47; 90%) in case reports, followed by tadalafil (n=4; 8%) and udenafil (n=1; 2%).<br>Type of outcome measures: All studies reported the risk of developing NAION with PDE5 inhibitors exposure. In case reports, the unit of analysis was each case report.	factors to develop NAION and medical history were recorded in three studies. In two studies, the PDE5 inhibitors were specified to vardenafil, tadalafil and sildenafil.<br>Three series of case reports comprising 22 case reports along with 28 case reports describing the development of NAION when the patient was exposed to a PDE5 inhibitor were identified. Eighteen case reports were from US. A single publication reported 10 case reports from Saudi Arabia. A total of 50 patients exposed to a PDE5 inhibitor with NAION were described in the literature. Forty-five (90%) patients were men. The average age of the patients were 52.5 years old (min= 7 months; max= 76). Twelve (23%) patients had not risk factors to develop NAION. Hypertension (n=15; 30%), diabetes mellitus (n=12; 24%) and dyslipidemia (n=10; 20%) were the most described risk factors. Thirty-nine (78%) case reports described patients treated for erectile dysfunction, and five (10%) case reports described patients treated for pulmonary arterial hypertension. Sildenafil was the PDE5 inhibitor most reported (n=45; 90%) in case reports, followed by tadalafil (n=4; 8%) and udenafil (n=1; 2%). The characteristics of case reports are described in Table 2.<br>In VigiBase, 6692 spontaneous reports on the SOC 'Eye disorders' were identified (Appendix 3). Of these, 608 belong to the PT 'Optic ischaemic neuropathy'.
Outcome analysis	Observational studies: Treatment with PDE5 inhibitors are not associated with an increased risk of NAION (OR 1.16; 95% CI 0.89, 1.52, p = 0.046; I2 = 62.6%) (Figure 3; Table 1).<br>Two case-crossover studies evaluated the association of intermittent use of PDE5 inhibitors and development of NAION. Both studies examined the risk of NAION associated with PDE5 inhibitors exposure within 5 half-lives compared with a more prior time period. The results showed that there is an	Observational studies: Treatment with PDE5 inhibitors are not associated with an increased risk of NAION (OR 1.16; 95% CI 0.89, 1.52, p = 0.046; I2 = 62.6%) (Figure 3; Table 1).<br>Two case-crossover studies evaluated the association of intermittent use of PDE5 inhibitors and development of NAION. Both studies examined the risk of NAION associated with PDE5 inhibitors exposure within 5 half-lives compared with a more prior time period. The results showed that there is an increased risk of NAION within five half-lives of PDE5 inhibitors use (OR

Step/ Review	A – Cochrane Collaboration	B – Centre for Reviews and Dissemination
	increased risk of NAION within five half-lives of PDE5 inhibitors use (OR 2.20; 95% CI 1.29, 3.76; p = 0.922; I2 = 0%) (Figure 3; Table 1).<br>Nathoo et al (2015), a retrospective nested case-control study, compared the risk of NAION in individuals exposed to PDE5 inhibitors to controls. The results were not statistically significant and concluded that there is not any association between PDE5 inhibitors exposure and NAION (OR 0.96 95% CI 0.75, 1.23) (Figure 3; Table 1). An identical result was achieved by Margo and French (2007) (OR 1.02; 95% CI 0.92, 1.13) (Figure 3; Table 1).<br>Sensitive analysis: The risk of NAION changed when the analysis included both definitive and possible cases of NAION (OR 1.28; 95% CI 0.95, 1.73; p = 0.012; I2 = 72.4%) (Figure 4).<br>Case reports: In the total of case reports, the administration of PDE5 inhibitors always precedes an event of NAION. A regular administration (≥ 2 months) of PDE5 inhibitors was observed in 25 (48%) case reports, whereas a recent administration was identified in 22 (42%) case reports. From the cases where a regular administration was reported, five patients admitted to double or triple the dose of PDE5 inhibitors. In general, the doses administered to each patient were within the approved. The majority of the cases reported the development of NAION in one eye (right eye = 22; 42%; left eye = 17; 33%). The characteristics and results of case reports are described in Table 2.<br>Spontaneous reports: "Optic ischaemic neuropathy", including NAION, was most reported with sildenafil (n=496), followed by tadalafil (n=79) and vardenafil (n=33) (Table 3).	2.20; 95% CI 1.29, 3.76; p = 0.922; I2 = 0%) (Figure 3; Table 1). However, the risk is not statistically significant.<br>Nathoo et al (2015), a retrospective nested case-control study, compared the risk of NAION in individuals exposed to PDE5 inhibitors to controls. The results were not statistically significant and concluded that there is not any association between PDE5 inhibitors exposure and NAION (OR 0.96 95% CI 0.75, 1.23) (Figure 3; Table 1). An identical result was achieved by Margo and French (2007) (OR 1.02; 95% CI 0.92, 1.13) (Figure 3; Table 1).<br>Sensitive analysis: The risk of NAION did not change when the analysis included both definitive and possible cases of NAION (OR 1.28; 95% CI 0.95, 1.73; p = 0.012; I2 = 72.4%) (Figure 3).<br>Case reports: In the total of case reports, the administration of PDE5 inhibitors always precedes an event of NAION. A regular administration (≥ 2 months) of PDE5 inhibitors was observed in 24 (48%) case reports, whereas a recent administration was identified in 22 (44%) case reports. From the cases where a regular administration was reported, four patients admitted to double or triple the dose of PDE5 inhibitors. In general, the doses administered to each patient were within the approved. The majority of the cases reported the development of NAION in one eye (right eye = 22; 44%; left eye = 17; 34%). The results of case reports are described in Table 2.<br>Spontaneous reports: "Optic ischaemic neuropathy", including NAION, was most reported with sildenafil (n=496), followed by tadalafil (n=79) and vardenafil (n=33).
Quality assessment	All case reports were assessed for bias (Appendix 4 – Characteristics of included studies). Despite a plausible biological mechanism can explain the development of NAION associated with PDE5 inhibitors exposure, the results of the observational studies evaluating the risk of such association were not significant. Therefore, none of the case reports have a good predictive value and causality, and cannot be used to demonstrate such association.<br>The risk of bias of each observational study was also assessed (Figure 2). The results are as the follows: *bias due to confounding* - One observational study was assessed as having critical risk of bias. No one of the confounders were	The full description of the methodological quality assessment was described in Appendix 4.<br>The methodological quality was assessed as good for three observational studies and fair for one observational study (Table 4). The study of Margo and French (2007) failed to report clearly the objective of the study. In the four observational studies, the patients were not blind to the exposure, neither the people who measure the outcomes. There was not randomization in any of the studies. The sample size was not estimated in any of the studies.

Step/ Review	A – Cochrane Collaboration	B – Centre for Reviews and Dissemination
	controlled. The other three studies were assessed as serious risk of bias; *bias in selection of the participants into the study* - In three studies, the selection process was strongly related with the intervention and the outcome. In the other study, the selection process only depended on outcome; *bias in classification of interventions* - All studies were assessed as low risk of bias. The intervention was well defined at the start of the study; *bias due to deviations from intended interventions* - All studies were assessed as low risk of bias. As observational studies, all deviations in study reflected the usual practice; *bias due to missing data* - All studies were assessed as low risk of bias. Data from the studies were complete; *bias in measurement of outcomes*: All studies were assessed as low risk of bias. The methods of assessment were comparable across intervention groups; *bias in selection of the reported result*: The studies did not provide sufficient information to evaluate this risk of bias.	For all case reports, a questionnaire was answered (Appendix 4). The exposure precedes the outcome. In some cases, the exposure was prolonged (≤ 2 months). For one case report, the dose was over those described in the Summary of Product Characteristics. The majority of patients had risk factors to develop NAION. Insufficient or unclear data on discontinuation and rechallenge was observed in the majority of case reports. In general, there are other factors that can explain the development of NAION.
Discussion	<u>Summary of main results</u>: Some observational studies studied the association of PDE5 inhibitors exposure and the development of NAION. However, their results were not statistically significant, even when compared the intermittent exposure of PDE5 inhibitors with exposure in a more previous time.<br>Several case reports described the development of NAION when the patient was taking a PDE5 inhibitor. The cases occurred mostly in men exposed to sildenafil for the treatment of erectile dysfunction. Almost 75% of patients had risk factors to develop NAION. In the majority of cases, the PDE5 inhibitor exposure was regular. NAION generally occurs in one eye.<br><u>Overall completeness and applicability of evidence</u>: This review included four observational studies. All of them have serious methodological issues, namely in assuring methods to avoid bias due to confounders, for example, determining the influence of risk factors to develop NAION or co-medications. Another critical issue was the selection of the participants into the study. In the included observational studies, the participants were selected according to the outcome and exposure, this is, the population was chosen according to the specific and pre-established aim leading to a risk of bias in the selection of participants. In the majority of the observational studies, the	<u>Principal findings</u>: Spontaneous reports were reported describing the development of NAION associated with PDE5 inhibitors exposure. Based on this data, in 2005, three regulatory agencies (European Medicines Agency (EMA), Food and Drug Administration (FDA), and Health Canada) issued a safety alert, warning healthcare professionals and consumers to be aware of visual changes related with sildenafil, tadalafil and vardenafil intake. The sections of the product label "Contraindications", "Warnings and Precautions", "Adverse reactions" and "Patient Counselling Information" were also updated.[16]<br>The association between the use of PDE5 inhibitors and the development of NAION is not yet established.[1,3,17,18] Several physiopathological hypotheses were studied. PDE5 inhibitors increase concentration of NO, prolonging vasodilation. This led to a rapid systemic hypotension, one of the risk factors of NAION.[17,18] PDE5 inhibitors may also have a role in the perfusion of optic nerve head, causing a local deregulation.[17,18] PDE6 enzyme is present in ocular blood vessels and have an important function in phototransduction. It is thought that PDE5 inhibitors also act on PDE6, being responsible for changes in color perception.[8,17] A pharmacological rationale can explain the development of NAION after PDE5 inhibitors exposure.

Step/ Review	A – Cochrane Collaboration	B – Centre for Reviews and Dissemination
	confounders were not controllable, since the population chosen was representative of the clinical practice.<br>The case reports also describe the events occurred in clinical practice. In general, the included case reports were well-described. However, some aspects such as causality result in higher risk in using this information to corroborate an association between PDE5 inhibitors use and NAION.<br>The data available on spontaneous reports was scarce, such as the therapeutic indication, patients' past medical history and risk factors, or case's causality assessment. Further, it was not possible to calculate incidences of NAION because no data of the exposed patients to each PDE5 inhibitor was measured.<br>Despite of the methodological problems observed on the available evidence, in 2005, the European Medicines Agency (EMA), the Food and Drug Administration (FDA) and Health Canada issued a safety alert based on spontaneous reports. The sections of the product label "Contraindications", "Warnings and Precautions", "Adverse reactions" and "Patient Counselling Information" were updated.[17]<br><u>Potential biases in the review process</u>: A protocol of this review was not previously published. The methodological quality level of the included evidence is low. Observational studies, case reports, and spontaneous reports are important tools in pharmacovigilance since they are useful to detect rare and/or long-term adverse reactions. However, observational designs are more likely to be subject of bias. The study search, selection and extraction process were systematic and independent, that should minimize bias.<br>Some sources of information are not available in our university (such as TRIP and Scopus databases) and they need the payment of a fee to access and perform searches.<br>The International Clinical Trials Register Platform and VigiBase are databases, developed and maintained by the World Health Organisation (WHO). The International Clinical Trials Register Platform contains trials registries from several worldwide data providers, such as ClinicalTrials.gov and EU Clinical Trials Register.[17] The VigiBase detain information reported to the WHO Programme for International Drug Monitoring from 120-member	In this review, in order to study such association, experimental and observational evidence was searched. We did not find experimental evidence studying this association. Nevertheless, four observational studies, along with 50 case reports and 608 spontaneous reports were identified.<br>According to the evidence found in this review, the cases occurred mostly in men exposed to sildenafil for the treatment of erectile dysfunction. NAION generally occurs in one eye after a regular PDE5 inhibitor exposure. The majority of the patients had other risk factors to develop NAION, such as hypertension. When pooled the results from the observational studies into a meta-analysis, the current available published evidence demonstrated to be insufficient to support an association between the development of NAION and PDE5 inhibitors exposure.<br><u>Comparison with other research</u>: Twenty-two reviews were identified in the search performed to this systematic review and meta-analysis. Of those, 12 (50%) reviewed specifically the association between PDE5 inhibitors exposure and the risk of NAION. Three systematic reviews identified some case reports and observational studies. Despite the present systematic review and meta-analysis has included more studies and case reports, the results of the previous published reviews were similar to those found in this systematic review.<br>One systematic review also performed a meta-analysis with observational studies.[18] An association between PDE5 inhibitors use and the development of NAION was also not found.[18] This review only included observational studies, excluding other type of observational data, such as case and series of case reports and spontaneous reports. This review included the four observational studies identified in our work along with the observational study by French and Margo (2008) which evaluated the association of PDE5 inhibitors plus organic nitrate or alfa-blockers and the development of NAION.[19] The study concluded that there was no increase in risk of NAION in men taking a PDE5 inhibitor with organic nitrates or an alfa-blocker compared with men taking PDE5 inhibitor alone.[19] This observational study was not included in the present systematic review since the aim of this study

Step/ Review	A – Cochrane Collaboration	B – Centre for Reviews and Dissemination
	countries.[19] The data provided by these two databases may not be completed and doesn't represent all worldwide data.<br>There was different designs and methodologies across the included observational studies. Such differences are usually associated with increased heterogeneity.[20] Therefore, the results should be interpreted cautiously. Nevertheless, case-crossover was the study design more properly used. In this design, each subject is his own control and is possible to estimate the risk of acute adverse events associated with intermittent drug exposures.[21]<br>Agreements and disagreements with other studies or reviews: Twenty-two reviews were identified in the search performed to this review. Of those, 12 (50%) reviewed specifically the association between PDE5 inhibitors exposure and the risk of NAION. Three systematic reviews identified some case reports and observational studies. Despite the present systematic review has included more studies and case reports, the results of the previous published reviews were similar to those found in this systematic review.<br>One systematic review also performed a meta-analysis with observational studies.[22] No association between PDE5 inhibitors use and the development of NAION was found.[22] This review included the observational study by French and Margo (2008) which evaluated the association of PDE5 inhibitors plus organic nitrate or alfa-blockers and the development of NAION.[23] The study concluded that there was no increase in risk of NAION in men taking a PDE5 inhibitor with organic nitrates or an alfa-blocker compared with men taking PDE5 inhibitor alone.[23] This observational study was not included in the present systematic review since it does not allow to measure the risk of PDE5 inhibitors alone.<br>One article analyzed the spontaneous reporting to the FDA of NAION associated with sildenafil, tadalafil and vardenafil. The first spontaneous report was reported in 1999 to sildenafil, one year after its marketing authorization. Since then, an increase in spontaneous reports were observed after FDA published the safety alert with cases describing such association. A more detailed and completed cases of NAION after PDE5 inhibitors intake was obtained through spontaneous reports systems.[24]	was to determine if the risk of developing NAION is increased with the co-medication of organic nitrate or alfa-blockers.<br>Another article analyzed the spontaneous reporting to the FDA of NAION associated with sildenafil, tadalafil and vardenafil. The first spontaneous report was reported in 1999 to sildenafil, one year after its marketing authorization. Since then, an increase in spontaneous reports were observed after FDA published the safety alert with cases describing such association. A more detailed and completed cases of NAION after PDE5 inhibitors intake was obtained through spontaneous reports systems.[20]<br>Strengths and weaknesses of the research: A key strength of this systematic review and meta-analysis is the combination of the published available evidence on clinical practice, including several types of evidence. Observational studies, case reports, and spontaneous reports are important tools in pharmacovigilance since they are useful to detect rare and/or long-term adverse reactions.<br>A protocol of this work was not previously published. Some sources of information are not available in our university (such as TRIP and Scopus databases) and they need the payment of a fee to access and perform searches. There are few studies evaluating the association between PDE5 inhibitors use and NAION. These studies have serious risk of bias and some limitations. Observational designs are likely to be subject of bias. There was different designs and methodologies across the included observational studies. Such differences are usually associated with increased heterogeneity.[21] Nevertheless, case-crossover was the study design more properly used. In this design, each subject is his own control and is possible to estimate the risk of acute adverse events associated with intermittent drug exposures.[21] Therefore, the results should be interpreted cautiously. The checklist used to assess the methodological quality is one of the checklists proposed by the CRD guidance for undertaking reviews in health care to assess non-randomized controlled trials.[13] However, this checklist may not provide detailed information on the insufficiencies of the studies. For instance, all the observational studies included are subject to exposure misclassification. Two observational studies

Step/ Review	A – Cochrane Collaboration	B – Centre for Reviews and Dissemination
		used data from clinical databases, one observational study applied a questionnaire to patients, and the other observational study did not specify the data source. Since PDE5 inhibitors are, generally, used periodically, data on exposure can be subject of exposure misclassification bias and/or recall bias. This bias and the low study power to detect the adverse drug reaction, may have led to the wide confidence intervals in the effect sizes for all studies. New large, prospective and comparative studies evaluating such association are needed.<br>A meta-analysis was conducted as recommended by the CRD guidance for undertaking reviews in health care.[13] Although a small number of studies was available, a quantitative synthesis allows to increase the sample size, narrow confidence interval and increase statistical power.[13] In this review, one of the observational studies detected an association between PDE5 inhibitors exposure and the development of NAION. However, when we pooled the results of all observational studies, the risk of developing this adverse drug reaction was not statistically significant. Thus, the result of the meta-analysis should be interpreted based on the limitations of the studies. We pooled the results according to the study design of the observational studies. We did not perform a meta-analysis to understand the influence of the risk factors, since this information is not clear in all the four observational studies.<br>The VigiBase database was developed and is maintained by the World Health Organization (WHO). The VigiBase detain information on spontaneous reported to the WHO Program for International Drug Monitoring from 120-member countries.[22] The data provided by this database may not be completed and doesn't represent all worldwide data. The data available on spontaneous reports was scarce, such as the therapeutic indication, patients' past medical history and risk factors, or case's causality assessment. Further, it was not possible to calculate incidences of NAION because no data of the exposed patients to each PDE5 inhibitor was measured.
Conclusion	<u>Implications for practice/ research</u>: There are few studies evaluating the association between PDE5 inhibitors use and NAION. These studies have serious risk of bias and several limitations. New large and comparative studies	<u>Recommendations/ implications for practice/ further research</u>: In light of the current available evidence, an association between PDE5 inhibitors exposure and NAION was not identified. However, since case and spontaneous reports

Step/ Review	A – Cochrane Collaboration	B – Centre for Reviews and Dissemination
	evaluating such association are needed. Despite the available evidence was scarce, a plausible mechanism can explain the development of NAION resultant from PDE5 inhibitors use. Additionally, several case reports and spontaneous reports have been published in literature. Some of them resulted in the generation of a safety alert from regulatory authorities. A close monitoring of the prescription of PDE5 inhibitors may be of great value in clinical practice.	have been reported, and in the light of a pharmacological rationale, a close monitoring is foreseen of great value.
References	1. Peeler C, Cestari DM. Non-Arteritic Anterior Ischemic Optic Neuropathy (NAION): A Review and Update on Animal Models. Semin Ophthalmol. 2016;31(1-2):99-106.<br>2. Kerr NM, Chew SS, Danesh-Meyer HV. Non-arteritic anterior ischaemic optic neuropathy: a review and update. J Clin Neurosci. 2009;16(8):994-1000.<br>3. Luneau K, Newman NJ, Biousse V. Ischemic optic neuropathies. Neurologist. 2008;14(6):341-54.<br>4. Mathews MK. Nonarteritic anterior ischemic optic neuropathy. Curr Opin Ophthalmol. 2005;16(6):341-5.<br>5. Huang SA, Lie JD. Phosphodiesterase-5 (PDE5) Inhibitors in the Management of Erectile Dysfunction. P T. 2013;38(7):407-19.<br>6. Frederick LR, Cakir OO, Arora H, Helfand BT, McVary KT. Undertreatment of erectile dysfunction: claims analysis of 6.2 million patients. J Sex Med. 2014;11(10):2546-2553.<br>7. Shamloul R, Ghanem H. Erectile dysfunction. Lancet. 2013;381(9861):153-65.<br>8. Ferguson JE 3rd, Carson CC 3rd. Phosphodiesterase type 5 inhibitors as a treatment for erectile dysfunction: Current information and new horizons. Arab J Urol. 2013 Sep;11(3):222-9.<br>9. Rew KT, Heidelbaugh JJ. Erectile Dysfunction. Am Fam Physician. 2016;94(10):820-827.<br>10. Koksal M, Ozdemir H, Kargi S, et al. The effects of sildenafil on ocular blood flow. Acta Ophthalmol Scand. 2005;83(3):355-9.<br>11. Montani D, Chaumais MC, Savale L, et al. Phosphodiesterase type 5 inhibitors in pulmonary arterial hypertension. Adv Ther. 2009;26(9):813-25.	1. Huang SA, Lie JD. Phosphodiesterase-5 (PDE5) Inhibitors in the Management of Erectile Dysfunction. P T. 2013;38(7):407-19.<br>2. Frederick LR, Cakir OO, Arora H, Helfand BT, McVary KT. Undertreatment of erectile dysfunction: claims analysis of 6.2 million patients. J Sex Med. 2014;11(10):2546-2553.<br>3. Shamloul R, Ghanem H. Erectile dysfunction. Lancet. 2013 Jan 12;381(9861):153-65.<br>4. Montani D, Chaumais MC, Savale L, et al. Phosphodiesterase type 5 inhibitors in pulmonary arterial hypertension. Adv Ther. 2009;26(9):813-25.<br>5. Barnes H, Brown Z, Burns A, et al. Phosphodiesterase 5 inhibitors for pulmonary hypertension. Cochrane Database of Systematic Reviews. 2017, Issue 4. Art. No.: CD012621.<br>6. Gacci M, Andersson KE, Chapple C, et al. Latest Evidence on the Use of Phosphodiesterase Type 5 Inhibitors for the Treatment of Lower Urinary Tract Symptoms Secondary to Benign Prostatic Hyperplasia. Eur Urol. 2016;70(1):124-133.<br>7. Giuliano F, Ückert S, Maggi M, et al. The mechanism of action of phosphodiesterase type 5 inhibitors in the treatment of lower urinary tract symptoms related to benign prostatic hyperplasia. Eur Urol. 2013;63(3):506-16.<br>8. Ferguson JE 3rd, Carson CC 3rd. Phosphodiesterase type 5 inhibitors as a treatment for erectile dysfunction: Current information and new horizons. Arab J Urol. 2013;11(3):222-9.

Step/ Review	A – Cochrane Collaboration	B – Centre for Reviews and Dissemination
	12. Barnes H, Brown Z, Burns A, et al. Phosphodiesterase 5 inhibitors for pulmonary hypertension. Cochrane Database of Systematic Reviews 2017, Issue 4. Art. No.: CD012621.<br>13. Gacci M, Andersson KE, Chapple C, et al. Latest Evidence on the Use of Phosphodiesterase Type 5 Inhibitors for the Treatment of Lower Urinary Tract Symptoms Secondary to Benign Prostatic Hyperplasia. Eur Urol. 2016;70(1):124-133.<br>14. Giuliano F, Ückert S, Maggi M, et al. The mechanism of action of phosphodiesterase type 5 inhibitors in the treatment of lower urinary tract symptoms related to benign prostatic hyperplasia. Eur Urol. 2013;63(3):506-16.<br>15. Liu B, Zhu L, Zhong J. The Association Between Phosphodiesterase Type 5 Inhibitor Use and Risk of Non-Arteritic Anterior Ischemic Optic Neuropathy: A Systematic Review and Meta-Analysis. Sex Med. 2018;6(3):185-192.<br>16. DerSimonian R, Laird N. Meta-analysis in clinical trials. Control Clin Trials 1986;7:177–188.<br>17. Penedones A, Mendes D, Alves C, Batel Marques F. Drug-induced ocular adverse reactions: review of the safety alerts issued during the last decade. J Ocul Pharmacol Ther. 2015;31(5):258-68.<br>18. World Health Organisation. International Clinical Trials Registry Platform Search Portal. 2018. Available at http://apps.who.int/trialsearch/ (last accessed December 14, 2018).<br>19. World Health Organisation. VigiAccess. 2018. Available at http://www.vigiaccess.org/ (last accessed December 14, 2018).<br>20. Alves C, Marques FB, Macedo AF. Drug-safety alerts issued by regulatory authorities: usefulness of meta-analysis in predicting risks earlier. Eur J Clin Pharmacol. 2014;70(6):745-56.<br>21. Strom BL: Novel Approaches to Pharmacoepidemiology Study Design and Statistical Analysis; in Strom BL, Kimmel SE (eds): Textbook of Pharmacoepidemiology. West Sussex, John Wiley & Sons, Ltd., 2006, pp 383-395.	9. Peeler C, Cestari DM. Non-Arteritic Anterior Ischemic Optic Neuropathy (NAION): A Review and Update on Animal Models. Semin Ophthalmol. 2016;31(1-2):99-106.<br>10. Kerr NM, Chew SS, Danesh-Meyer HV. Non-arteritic anterior ischaemic optic neuropathy: a review and update. J Clin Neurosci. 2009;16(8):994-1000.<br>11. Luneau K, Newman NJ, Biousse V. Ischemic optic neuropathies. Neurologist. 2008 Nov;14(6):341-54.<br>12. Mathews MK. Nonarteritic anterior ischemic optic neuropathy. Curr Opin Ophthalmol. 2005;16(6):341-5.<br>13. Centre for Reviews and Dissemination, University of York. CRD's guidance for undertaking reviews in health care. https://www.york.ac.uk/media/crd/Systematic_Reviews.pdf. Accessed 09.01.2019.<br>14. Downs SH, Black N. The feasibility of creating a checklist for the assessment of the methodological quality both of randomised and non-randomised studies of health care interventions. J Epidemiol Community Health. 1998;52(6):377-84.<br>15. DerSimonian R, Laird N. Meta-analysis in clinical trials. Control Clin Trials. 1986;7:177–188.<br>16. Penedones A, Mendes D, Alves C, Batel Marques F. Drug-induced ocular adverse reactions: review of the safety alerts issued during the last decade. J Ocul Pharmacol Ther. 2015;31(5):258-68.<br>17. Koksal M, Ozdemir H, Kargi S, et al. The effects of sildenafil on ocular blood flow. Acta Ophthalmol Scand. 2005;83(3):355-9.<br>18. Liu B, Zhu L, Zhong J. The Association Between Phosphodiesterase Type 5 Inhibitor Use and Risk of Non-Arteritic Anterior Ischemic Optic Neuropathy: A Systematic Review and Meta-Analysis. Sex Med. 2018;6(3):185-192.<br>19. French DD, Margo CE. Post-marketing surveillance of ischaemic optic neuropathy in male veterans co-prescribed phosphodiesterase-5 inhibitors with organic nitrates or alpha-blockers. Drug Saf 2008;31(3):241-7.

Step/ Review	A – Cochrane Collaboration	B – Centre for Reviews and Dissemination
	22. Liu B, Zhu L, Zhong J, et al. The Association Between Phosphodiesterase Type 5 Inhibitor Use and Risk of Non-Arteritic Anterior Ischemic Optic Neuropathy: A Systematic Review and Meta-Analysis. Sex Med. 2018;6(3):185-192.<br>23. French DD, Margo CE. Post-marketing surveillance of ischaemic optic neuropathy in male veterans co-prescribed phosphodiesterase-5 inhibitors with organic nitrates or alpha-blockers. Drug Saf 2008;31(3):241-7.<br>24. Pomeranz HD. Cases of Ischemic Optic Neuropathy Associated With Phosphodiesterase-5 Inhibitor Use Reported to the Food and Drug Administration Adverse Event Reporting System. J Neuroophthalmol. 2016;36(2):221-2.	20. Pomeranz HD. Cases of Ischemic Optic Neuropathy Associated With Phosphodiesterase-5 Inhibitor Use Reported to the Food and Drug Administration Adverse Event Reporting System. J Neuroophthalmol. 2016;36(2):221-2.<br>21. Strom BL: Novel Approaches to Pharmacoepidemiology Study Design and Statistical Analysis; in Strom BL, Kimmel SE (eds): Textbook of Pharmacoepidemiology. West Sussex, John Wiley & Sons, Ltd., 2006, pp 383-395.<br>22. World Health Organization. VigiAccess. 2018. http://www.vigiaccess.org/. Accessed 14.12.2018.
Funding	Financial Support: None.<br>Conflict of Interest: No conflicting relationship exists for any author.	Financial Support: None.<br>Conflict of Interest: No conflicting relationship exists for any author.
Appendix	Appendix 1 - Search strategy;<br>Appendix 2 - List of included and excluded studies;<br>Appendix 3 - Vigibase results;<br>Appendix 4 - Characteristics of studies and quality assessment results.	Appendix 1 - Search strategy;<br>Appendix 2 - List of included and excluded studies;<br>Appendix 3 - Vigibase results;<br>Appendix 4 - Quality assessment results.
Tables	Table 1 – Observational studies summary.<br>Table 2 – Characteristics and results of case reports.<br>Table 3 – Spontaneous reports of PDE5 inhibitors registered in VigiBase.<br>Table 4 – Scores of the methodological quality assessment of the observational studies.	Table 1 – Observational studies summary.<br>Table 2 – Characteristics and results of case reports.<br>Table 3 – Spontaneous reports of PDE5 inhibitors registered in VigiBase.<br>Table 4 – Scores of the methodological quality assessment of the observational studies.
Figures	Figure 1 – PRISMA flow chart of search strategy and study selection.<br>Figure 2 – Odds Ratios and 95% Confidence Intervals for definitive cases of NAION associated with PDE5 inhibitors.<br>Figure 3 – Odds Ratios and 95% Confidence Intervals for definitive and possible cases of NAION associated with PDE5 inhibitors.	Figure 1 – PRISMA flow chart of search strategy and study selection.<br>Figure 2 – Odds Ratios and 95% Confidence Intervals for definitive cases of NAION associated with PDE5 inhibitors.<br>Figure 3 – Odds Ratios and 95% Confidence Intervals for definitive and possible cases of NAION associated with PDE5 inhibitors.

* Databases not accessible to the authors of the reviews.
